# Perceived parental differential treatment and internet addiction among Chinese adolescents: the mediating role of self-esteem and the moderating role of fairness perception

**DOI:** 10.3389/fpsyg.2026.1916688

**Published:** 2026-10-05

**Authors:** Xinxin Shi, Junting Hou, Zhiyun Wang

**Affiliations:** School of Education and Cognitive Sciences, Zhengzhou University, Zhengzhou, China

**Keywords:** adolescents, fairness perception, internet addiction, perceived parental differential treatment, self-esteem

## Abstract

This study explores the correlational associations between perceived parental differential treatment (PDT) and Internet addiction (IA) among Chinese adolescents, as well as the mediating role of self-esteem and the moderating role of fairness perception. A sample of 426 non-only children aged 9–19 years (*M* = 13.30, *SD* = 2.00) was recruited from five schools in Hebei, Henan, Jilin, and Shandong provinces using cluster-based convenience sampling. Participants completed self-report questionnaires, including the Sibling Experience Questionnaire, Young’s Internet Addiction Diagnostic Scale, Rosenberg Self-esteem Scale, and the Fairness Perception Scale. The results indicated that: (1) PDT (perceived self-favoritism) was significantly negatively correlated with IA; (2) self-esteem served as a full mediator in the correlational pathway linking PDT and IA; (3) fairness perception significantly moderated the association between paternal PDT and IA. The negative association between paternal PDT and IA emerged only at high levels of fairness perception, though this conditional association was marginally significant. These findings highlight the potential value of positive self-esteem and perceived fairness for understanding IA among adolescents in multi-child families, providing both theoretical and practical implications for family-based interventions.

## Introduction

With the rapid advancement of digital technology, China has witnessed a surge in adolescent internet users. According to the 55th Statistical Report on Internet Development in China (China Internet Network Information Center [CNNIC], 2025), the number of internet users aged 10–19 reached 144 million in 2024, accounting for 13% of the total internet user base. While the internet provides adolescents with unprecedented access to information, excessive and uncontrolled use can lead to internet addiction (IA). As defined by Young (1996), this form of behavioral addiction is marked by compulsive online behaviors, abandonment of daily routines and duties, withdrawal reactions upon internet deprivation, as well as detrimental outcomes regarding individuals’ mental health and social adaptation.

Adolescents experiencing internet addiction have precipitated a notable public health crisis, given its association with numerous detrimental outcomes such as impaired academic functioning (Mohamed and Bernouss, 2020), increased risk of depression and anxiety (Ergün et al., 2025), and strained family and peer relationships (Shek et al., 2019). Given these far-reaching impacts, identifying the risk and protective factors associated with adolescent internet addiction has become a critical focus of research and intervention efforts.

Family environment, particularly parenting practices, is widely recognized as a key determinant of adolescent internet use behaviors (Li et al., 2018). In recent years, China’s fertility policy has undergone significant adjustments, with the implementation of the universal two-child policy in 2016 and the three-child policy in 2021, resulting in a substantial increase in multi-child families. This demographic shift has drawn attention to parental differential treatment. Defined as uneven allocation of affection, material resources, and disciplinary practices across siblings, this construct describes a widespread yet insufficiently examined familial dynamic within Chinese cultural settings (Zhang and Cao, 2021).

Parental differential treatment differs from general parenting styles in that it is inherently comparative, involving parents treating siblings differently rather than adopting a uniform approach to all children (Daniels and Plomin, 1985). Unlike Western cultures, where individualism emphasizes equal treatment of children, collectivism culture acknowledges hierarchical family roles (e.g., “respect for elders and care for juniors”) and contextualizes fairness within family dynamics, which may normalize certain forms of PDT (Luo et al., 2020). For example, parents may allocate more resources to younger children due to their greater needs, or show preferential treatment to a child with specific talents or challenges, and such practices may be viewed as “fair” within the cultural framework of “teaching according to aptitude” (Zhang and Cao, 2021). Confucian cultural concepts may further shape the manifestations of PDT, the ethical norm of “ordering the eldest and the youngest” may lead parents to have higher academic expectations of their older children and provide more life care for their younger ones (Chen and Shi, 2017). Although objective disparities in parental treatment exist, their developmental impacts largely depend on adolescents’ subjective interpretations of such differences.

Gender disparities constitute another influential factor shaping PDT. For mixed-gender sibling dyads consisting of one son and one daughter, parents generally allocate greater emotional support to daughters relative to sons. By contrast, households with two male siblings tend to witness harsher disciplinary regulation from caregivers (Jensen et al., 2015). These patterns all reflect the traditional parenting logic of “young people require care,” “elders bear familial responsibilities,” and gendered expectations for sons and daughters. Adolescents may perceive such divergent parental treatment yet view these arrangements as understandable within the family context.

Despite the prevalence of PDT in multi-child families, its association with adolescent IA remains poorly understood. Existing research on parenting and IA has primarily focused on general parenting styles (e.g., authoritarian vs. authoritative; Liu and Li, 2017) or specific behaviors (e.g., parental phubbing; Zhou et al., 2022), with limited attention to the comparative nature of PDT. Moreover, the mechanisms underlying the potential link between PDT and IA, such as psychological variables that may mediate or moderate this relationship, have yet to be systematically explored.

To address these gaps, the present study proposes a moderated mediation model to examine: (1) the direct association between PDT and IA; (2) the mediating role of self-esteem in this relationship; and (3) the moderating role of fairness perception in both the direct and indirect paths. By investigating these questions, we aim to advance understanding of how family processes influence adolescent IA in the unique cultural context of contemporary Chinese multi-child families and to provide evidence-based insights for prevention and intervention strategies.

### Perceived parental differential treatment and internet addiction

Parental differential treatment refers to parents’ unequal behaviors toward siblings across domains such as affection, instrumental support, and discipline (Jensen and Whiteman, 2014). The concept was first formalized by Daniels and Plomin (1985), who argued that even within the same family, siblings often experience distinct parenting environments due to the interaction of parental resources (e.g., time, energy, emotional availability) and child-specific characteristics (e.g., temperament, gender, birth order, academic performance; McHale et al., 2000).

In Chinese families, PDT is shaped by both cultural norms and structural factors. Traditional values such as “preferring sons over daughters,” though declining, may still influence parental resource allocation in some regions (Tucker et al., 2003). Additionally, the emphasis on “elders caring for juniors” may lead parents to prioritize younger siblings’ needs, resulting in differential treatment that is perceived as legitimate within the family (Luo et al., 2020). Zhang and Cao (2021) noted that PDT in Chinese two-child families is often justified by parents as “adapting to each child’s unique needs,” further highlighting the cultural embeddedness of this phenomenon. It should be noted that these broad cultural-norm-related influences are not uniform across Chinese families. Perceptions toward PDT can vary by region, socioeconomic status, urban-rural background, family structure, and generational shifts. Cultural norms represent societal ideals rather than universal lived experiences for all adolescents.

Research on family systems theory suggests that PDT due to an unequal distribution of parental energy is significantly higher in families where there is an age difference of more than 5 years between siblings, and that such differentiation is positively correlated with the frequency of sibling conflict among adolescents (Yang et al., 2022). Following the implementation of the “double-only two-child” policy, the first child experienced the transition from being an only child to becoming an older brother or sister, made them more sensitive to parental favoritism than younger individuals who were born into families with multiple siblings. They were also experience a sense of deprivation due to the “sudden change of roles,” suggesting that the effects of PDT can manifest differently in response to changes in family structure (Luo et al., 2020).

Empirical research consistently demonstrates that PDT has significant implications for child development, although the effects depends on the “direction” of favoritism (i.e., whether the child perceives themselves as favored or disfavored relative to siblings). Perceived favoritism toward siblings is associated with a range of negative outcomes, including increased sibling conflict (Hamwey and Whiteman, 2021), lower life satisfaction (Suitor et al., 2017), and higher levels of internalizing problems such as anxiety and depression (Jeannin and Van Leeuwen, 2015). Conversely, favoritism toward the self is linked to fewer externalizing behaviors (e.g., delinquency; Jensen and Whiteman, 2014) and higher self-reported wellbeing (Suitor et al., 2017), suggesting that being the “preferred” child may confer psychological benefits. However, favored children’s elevated self-esteem may be merely “conditioned self-esteem,” making them vulnerable to identity crises when parental attention shifts (McHale et al., 2000). Although self-favoritism offers short-term protection, it also brings risks including sibling jealousy, guilt, and pressure to maintain parental approval. As Jensen et al. (2018) demonstrated, PDT can outweigh general parenting quality; even positive parenting can be harmful if adolescents perceive receiving fewer resources than siblings. These observations are largely from Western samples, and the present study aims to examine these dynamics within Chinese multi-child families.

In research on internet addiction (IA), adverse parenting behaviors including overcontrolling parenting, emotional neglect, and erratic disciplinary strategies have been widely verified as salient risk factors (Liu and Li, 2017). PDT may influence IA through similar mechanisms, but its effects are likely to be conditional on the direction of favoritism. Adolescents who perceive themselves as disfavored may experience emotional neglect or frustration, leading them to seek compensation or escape through internet use (Karaer and Akdemir, 2019). For example, disfavored children may turn to online gaming or social media to fulfill unmet needs for attention, validation, or social connection (Ching and Tak, 2017). In terms of psychosocial mechanisms, PDT essentially reflects an uneven distribution of parenting resources among siblings. Unfavored children often experience emotional deprivation and control repression, and their needs for belonging and autonomy are not met within the family. Consequently, they seek alternative satisfactions online (Deci and Ryan, 2000; Subrahmanyam et al., 2001). As proposed in problem behavior theory Jessor’s (1987), adolescents experiencing chronic parental neglect tend to build virtual interpersonal bonds online or seek a sense of achievement from video games to offset setbacks in offline life, a pathway that ultimately elevates their vulnerability to internet addiction. In contrast, adolescents who feel favored may have stronger offline social support and psychological security, reducing their reliance on the internet as a source of comfort or validation (Jensen and Whiteman, 2014; Karaer and Akdemir, 2019).

Importantly, although self-favoritism may confer such adaptive benefits under typical conditions, theoretical alternatives remain. Excessive self-favoritism could bring about sibling-related jealousy, psychological dependency, entitlement, and poor autonomy development, which may conversely raise the risk of problematic internet use (Jensen et al., 2018). Nonetheless, extreme levels of perceived parental differential treatment are uncommon in community-based adolescent samples. Drawing on existing literature emphasizing the protective function of self-favoritism in ordinary contexts, we propose the following directional hypotheses, while these competing adverse pathways are reserved for future investigation. Thus, we hypothesize:

H1: Perceived parental differential treatment (favoritism toward the self) is negatively associated with adolescent internet addiction.

### The mediating role of self-esteem

Self-esteem, defined as an individual’s global evaluation of their self-worth and competence (Rosenberg, 1965), is a core psychological resource that shapes adaptive behaviors and emotional regulation. High self-esteem is characterized by a positive self-concept, resilience in the face of stress, and confidence in one’s ability to cope with challenges. In contrast, low self-esteem is linked to emotional distress, self-doubt, and a tendency to engage in maladaptive coping strategies (e.g., avoidance, substance use; Wood and Forest, 2016).

Parental differential treatment is a significant predictor of self-esteem development. Children who receive more parental affection, praise, and autonomy (i.e., are favored) tend to internalize these positive evaluations, developing higher self-esteem (Pinquart and Gerke, 2019). Sheehan and Noller (2002) found that adolescents who perceived greater parental care and less control reported higher self-esteem, as they felt valued and accepted by their parents. Conversely, disfavored children receive diminished parental affection alongside heightened criticism and restrictive supervision. Such youth tend to exhibit poorer self-esteem, a pattern attributed to social comparison with siblings (Feinberg et al., 2000) and subjective feelings of parental rejection (Shebloski et al., 2005). For example, Zervas and Sherman (1994) demonstrated that adolescents who perceived parental favoritism toward their siblings reported significantly lower self-esteem than those who felt equally treated.

According to social comparison theory, adolescents evaluate themselves by comparing their parents’ emotional investment and resource allocation toward them versus their siblings (Kowal and Kramer, 1997). Neglected adolescents often attribute differential treatment to their own inadequacies, and prolonged self-denial can lead to significantly lower self-esteem. Furthermore, attachment theory reveals that consistent and stable emotional responses from parents form the basis of secure attachments. However, PDT disrupts such relational equilibrium. Children who receive consistent preferential care tend to form anxious attachment patterns, relying on persistent special parental favor to sustain their self-worth. In contrast, youth facing long-term neglect adopt avoidant attachment styles, they shield themselves via emotional disengagement, a mechanism that impedes healthy self-esteem formation (Bowlby, 1969).

Self-esteem, in turn, is inversely related to IA. Adolescents with low self-esteem may use the internet to compensate for unmet psychological needs, such as the need for belonging, competence, or self-worth (Shi et al., 2017). Online environments facilitate identity exploration, the pursuit of social recognition, and temporary relief from negative self-evaluations. These dimensions draw adolescents with low self-esteem toward problematic over-engagement with the internet (Ayas and Horzum, 2013). In contrast, high self-esteem acts as a protective factor. Adolescents who hold positive self-perceptions demonstrate stronger capacity to manage online behaviors, given their solid confidence in real-world interpersonal connections and stress coping skills (Andreassen et al., 2017).

Thus, the above results indicate that PDT exerts an indirect effect on IA via self-esteem. Perceived favoritism enhances individuals’ self-esteem, which in turn reduces the risk of IA. Thus, we hypothesize:

H2: Self-esteem mediates the relationship between PDT (favoritism toward the self) and internet addiction.

### The moderating effect of fairness perception

Fairness perception refers to an individual’s subjective judgment of whether a situation or treatment is just or equitable (Li et al., 2003). In the context of PDT, fairness perception is not about absolute equality but about whether the between-sibling differential treatment is perceived as legitimate, reasonable, or justified (Gozu and Newman, 2020). For example, a child may accept that a younger sibling receives more parental attention if they understand it as necessary for the sibling’s development, but resent the same treatment if they perceive it as arbitrary or unjust.

Based on the equity theory, individuals evaluate relationships by comparing their own outcomes (e.g., parental affection) to their inputs (e.g., effort to please parents) relative to others (Hatfield et al., 1978). When outcomes are perceived as inequitable, individuals experience psychological distress (e.g., anger, resentment), which amplifies the negative effects of adverse experiences. In the context of PDT, perceived unfairness may exacerbate the harm of disfavored (e.g., lowering self-esteem further) or weaken the benefits of favoritism (e.g., reducing the positive impact of being favored). Conversely, high fairness perception may buffer against the negative effects of PDT or strengthen its positive effects (Kowal et al., 2004). For instance, Kowal and Kramer (1997) found that approximately one-third of children perceived differential parental treatment; however, 75% of these children did not perceive it as “unfair,” meaning that their sibling relationships were not significantly affected. In the families with autistic or disabled children, parents tend to allocate more resources to caring for children with special needs. However, other children who understand that this difference is based on actual need do not experience negative emotions (Boll et al., 2005; Chan and Goh, 2014).

In collectivism culture, fairness is often contextualized within family roles and hierarchical norms (Luo et al., 2020). Older siblings, socialized to “set an example” and care for younger siblings, may be more likely to perceive PDT as fair, for example, viewing a younger sibling’s preferential treatment as legitimate due to their age (Suitor et al., 2017). This cultural framing may shape how adolescents evaluate PDT, making fairness perception a particularly important moderator in multi-child families. Chen (2005) points out that equity is not an absolute “average distribution,” but rather a relative balance based on differences in individual needs. The core of this concept lies in whether the distribution of resources and emotional inputs aligns with individual perceptions of what is “reasonable.” This viewpoint is consistent with the traditional Chinese educational philosophy of “teaching according to aptitude.”

Thus, identical parental differential treatment may produce divergent psychological outcomes, depending on how adolescents subjectively interpret the reasonableness of such treatment. Fairness perception shapes the psychological meaning attributed to PDT, rather than simply being a psychological outcome triggered by differential treatment itself. Adolescents may experience comparable levels of PDT yet judge it as reasonable or unjust based on family context, cultural norms and situational considerations. These interpretive judgments amplify or buffer the developmental consequences of PDT, altering the magnitude of PDT’s predictive effects on self-esteem and internet addiction.

Accordingly, fairness perception is conceptualized as a moderator. Favoritism perceived as reasonable may enhance family security and belonging, benefiting adolescents’ self-esteem while reducing compensatory online behaviors. By contrast, differential treatment viewed as unfair may trigger family dissatisfaction, impair self-esteem, and promote escape-oriented internet use. Based on this, we hypothesize:

H3: Fairness perception moderates the relationship between PDT and self-esteem, such that the positive association between favoritism toward the self and self-esteem is stronger when fairness perception is high.

H4: Fairness perception moderates the relationship between PDT and internet addiction, such that the negative association between favoritism toward the self and internet addiction is stronger when fairness perception is high.

### The present study

This study aims to test the proposed moderated mediation models (see Figure 1), with a focus on distinguishing between paternal and maternal PDT. Previous research suggests that fathers and mothers may play distinct roles in shaping adolescent outcomes. Caregiving behaviors from mothers display closer correlations with adolescents’ self-esteem (Furnham and Cheng, 2000), whereas paternal parenting exerts more pronounced influences on behavioral outcomes including IA (Shek et al., 2019). By examining paternal and maternal PDT separately, we seek to clarify whether the proposed mechanisms operate differently based on parental gender.

**FIGURE 1:**
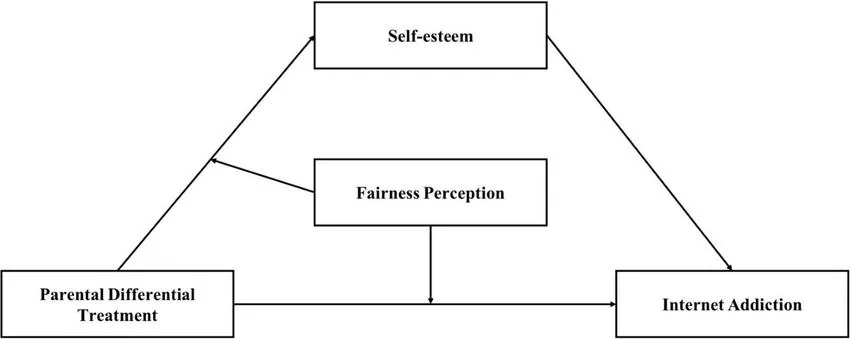
Hypothesized model.

Overall, this study contributes to the literature by: (1) investigating the understudied relationship between PDT and IA in Chinese multi-child families; (2) identifying self-esteem as a potential mediator, shedding light on the psychological processes linking PDT to IA; (3) examining fairness perception as a moderator, highlighting the role of subjective evaluations in shaping the effects of PDT; and (4) distinguishing paternal and maternal effects, addressing the gendered nature of parenting in Chinese culture. The findings will inform family-based interventions aimed at reducing adolescent IA, particularly in the context of China’s growing multi-child families.

## Methods

### Participants and procedures

Participants were recruited from 5 schools (two primary schools, two middle schools, and one high school) in Hebei, Henan, Jilin, and Shandong provinces using cluster-based convenience sampling. Schools were recruited based on the geographical accessibility of the research team rather than systematic representative sampling. All participating schools were located at county-level or above, and no rural schools were included in the present sample. Within each school, classes with varying academic performance levels were matched and selected as clusters. Inclusion criteria were: (1) being a non-only child (i.e., having at least one sibling); (2) aged 9–19 years; (3) able to read and complete questionnaires independently; and (4) capable of independently understanding and completing self-report questionnaires; students with functional difficulties that would prevent valid questionnaire completion were excluded. All eligible students in these selected classes were invited to participate.

A total of 487 adolescents were initially recruited. After data screening, 61 participants were excluded. These individuals had fully blank responses or only a few completed items for at least one core study variable. Among them, two had incomplete records for internet addiction and self-esteem, 32 for parental differential treatment, and 27 for fairness perception. The final sample included 426 valid responses, with an effective response rate of 87.5%. Comparisons between excluded (*n* = 61) and retained (*n* = 426) participants showed no significant differences in birth order, number of siblings, self-esteem, internet addiction, DTM, and DTF (all *p* > 0.05). Significant differences emerged for gender, age, school level, paternal and maternal fairness perception (all *p* < 0.05). Excluded participants were older, more boys, from higher school levels, and reported lower fairness perceptions of PDT. Notably, no group differences were observed for our primary outcome (internet addiction), mediator (self-esteem), and PDT exposure variables.

Demographic characteristics of the sample are as follows: 182 boys (42.7%) and 237 girls (55.6%), seven participants (1.6%) did not report gender; all valid participants fell within the predefined 9–19-year-old range (*M* = 13.30, SD = 2.00), including 131 primary-school students, 204 middle-school students, and 91 high-school students, including 363 (85.2%) from two-child families, 60 (14.1%) from three-child families, and 3 (0.7%) from four-child families. Sibling gender composition was coded based on each participant’s closest-in-age sibling, with valid data available for 371 participants: 76 (17.8%) brother-brother pairs, 89 (20.9%) sister-sister pairs, 94 (22.1%) brother-sister pairs, and 112 (26.3%) sister-brother pairs. The 55 missing cases for this variable resulted from participants not reporting their own gender or the gender of their closest-in-age sibling. Relative to this reference sibling, the mean age difference was 6.35 years (SD = 3.39); 153 (35.9%) participants identified as the older sibling, 218 (51.2%) as the younger sibling, and 55 (12.9%) could not be clearly classified as older or younger due to twins or unreported age information for the reference sibling.

Data collection was conducted in classrooms during regular school hours. Trained research assistants (graduate students in psychology) provided standardized verbal and written instructions, emphasizing that participation was voluntary and anonymous, and that responses would be used for research purposes only. Informed consent was obtained from both participants and their parents/guardians prior to data collection. Participants completed the questionnaires independently, with research assistants available to clarify questions if needed. The average completion time was 20 min.

The study protocol was reviewed and approved by Ethics Committee of the author’s university. To address potential common method variance (CMV) associated with self-report measures, we implemented procedural controls (e.g., anonymous data collection, clear instructions, participants were informed there were no right or wrong answers) and statistical controls (Harman’s single-factor test; Podsakoff et al., 2003). Exploratory factor analysis (EFA) of all items across scales revealed more than one principal component with an eigenvalue greater than 1, with the first factor explaining 24.78% of variance (below the 40% threshold), suggesting no severe common-method bias in this dataset.

### Measures

Perceived parental differential treatment: PDT in the current study refers to adolescents’ subjective appraisal of parental treatment relative to their closest-in-age sibling. PDT was assessed using the Chinese version of the Chinese Sibling Experience Questionnaire (Daniels and Plomin, 1985; Ong and Tan, 2018). The scale includes nine items measuring parental behaviors toward the participant versus their closest-in-age sibling, with separate ratings for fathers and mothers. Items cover two dimensions: affection (pride, interest, favoritism, enjoyment, and sensitivity) and control (strictness, punishment, blame, and discipline). Responses are on a five-point Likert scale (1 = applies more to my sibling, 5 = applies more to me). Control items were reverse-coded such that higher scores indicate greater favoritism toward the self. This scoring follows the original SIDE framework, yet it does not fully account for Chinese cultural contexts, where greater discipline may signal parental investment rather than disfavor. The scale has demonstrated good reliability and validity in Chinese samples (Shi et al., 2022). In this study, Cronbach’s α was 0.72 for both paternal and maternal subscales, indicating acceptable internal consistency.

Internet addiction: IA was measured using Young (1996) Internet Addiction Diagnostic Scale, a widely used 10-item instrument assessing symptoms of IA. Responses are binary (1 = yes, 0 = no), with higher total scores indicating more severe IA. The scale has been validated in Chinese adolescents (Li et al., 2018), Cronbach’s α was 0.79 in this study, indicating good reliability.

Self-esteem: Self-esteem was assessed using Rosenberg (1965) Self-Esteem Scale, a 10-item measure of global self-worth (e.g., “I feel that I am a person of worth, at lease on an equal basis with others”). Responses are rated on a four-point Likert scale (1 = very strongly disagree, 4 = very strongly agree), with higher total scores indicating higher self-esteem. The scale is widely used in Chinese samples (Shi et al., 2017) and demonstrated excellent reliability in the present study (Cronbach’s α = 0.84).

Fairness perception: Fairness perception regarding PDT was measured using a scale adapted from Gozu and Newman (2020). Participants rated the perceived legitimacy of PDT described in the Sibling Experience Questionnaire, evaluating whether such between-sibling differences were reasonable rather than judging absolute equal distribution of parental resources. Responses are on a five-point Likert scale (1 = very unfair, 5 = very fair), with higher scores indicating stronger perceived fairness. Separate scores were calculated for paternal and maternal treatment. The scale demonstrated excellent reliability in the present study (Cronbach’s α = 0.97 for both paternal and maternal subscales). This high coefficient raises potential concerns regarding item redundancy. Given that fairness items are derivative and one-to-one matched with PDT items, CFA-driven item trimming was not performed to preserve complete evaluation of PDT-related behaviors.

### Statistical analysis

Data were analyzed using SPSS 23.0. First, descriptive statistics (means, standard deviations) and bivariate correlations were computed to examine relationships between key variables. Next, hierarchical regression analysis was conducted to test the moderated mediation model using Hayes (2013) PROCESS macro (Model 8), which estimates indirect effects conditional on a moderator.

Gender, age, sibling gender composition, age space, and birth order were included as covariates in all analyses. All variables were standardized (z-scored) before analysis to facilitate the interpretation of interaction effects. Significance was determined using two-tailed tests with α = 0.05, and 95% bias-correlated confidence intervals (CIs) for indirect effects were computed using 5,000 bootstrapped samples.

## Results

### Preliminary analyses

Table 1 presents descriptive statistics and correlations. It revealed that both paternal and maternal PDT (favoritism toward the self) was negatively correlated with IA and positively correlated with self-esteem and fairness perception. Self-esteem was negatively correlated with IA. Fairness perception was negatively correlated with IA and positively correlated with self-esteem.

**TABLE 1:** Descriptive statistics and inter-correlations of the variables (*N* = 426).

Variables		M ± SD	6	7	8	9	10	11	12	13	14	15	16	17	18	19
1.Gender		1.57 ± 0.50	−0.19***	−0.06	0.06	−0.13**	−0.11*	−0.01	0.04	−0.03	−0.02	−0.002	−0.07	−0.02	−0.14**	−0.04
2.Age		13.30 ± 2.00	−0.12*	0.03	0.08	−0.13*	−0.08	0.01	0.05	0.01	0.02	0.04	0.02	0.02	−0.25***	0.23***
3. Gender compositions		2.65 ± 1.12	−0.06	−0.04	0.001	−0.06	−0.008	−0.03	0.01	0.02	0.02	0.04	0.01	0.02	−0.13	0.06
4. Age space		6.35 ± 3.39	0.07	0.04	−0.01	0.06	0.01	−0.03	−0.01	−0.02	0.002	0.04	0.01	0.01	−0.003	−0.02
5. Birth order		1.59 ± 0.49	0.14**	−0.14**	−0.18***	0.12*	−0.10	−0.13*	−0.15**	−0.10	−0.12*	−0.11*	−0.10	−0.12*	0.13*	−0.02
Perceived parental differential treatment	6. Paternal differential control	3.22 ± 0.60	1.00	–	–	–	–	–	–	–	–	–	–	–	–	–
	7. Paternal differential affection	2.95 ± 0.62	−0.21***	1.00	–	–	–	–	–	–	–	–	–	–	–	–
	8. Paternal differential treatment	2.87 ± 0.48	−0.71***	0.84***	1.00	–	–	–	–	–	–	–	–	–	–	–
	9. Maternal differential control	3.21 ± 0.55	0.59***	−0.10*	−0.40***	1.00	–	–	–	–	–	–	–	–	–	–
	10. Maternal differential affection	2.97 ± 0.62	−0.09	0.49***	0.40***	−0.23***	1.00	–	–	–	–	–	–	–	–	–
	11. Maternal differential treatment	2.89 ± 0.47	−0.37***	0.42***	0.51***	−0.69***	0.86***	1.00	–	–	–	–	–	–	–	–
Fairness perception	12. Paternal differential control	3.77 ± 0.95	−0.28***	0.39***	0.44***	−0.28***	0.20***	0.30***	1.00	–	–	–	–	–	–	–
	13. Paternal differential affection	3.82 ± 0.86	−0.18***	0.43***	0.41***	−0.18***	0.26***	0.29***	0.87***	1.00	–	–	–	–	–	–
	14. Paternal differential treatment	3.80 ± 0.85	−0.20***	0.38***	0.39***	−0.29***	0.32***	0.39***	0.93***	0.95***	1.00	–	–	–	–	–
	15. Maternal differential control	3.77 ± 0.97	−0.19***	0.31***	0.32***	−0.38***	0.32***	0.43***	0.87***	0.81***	0.93***	1.00	–	–	–	–
	16. Maternal differential affection	3.83 ± 0.88	−0.12*	0.31***	0.29***	−0.28***	0.41***	0.45***	0.78***	0.87***	0.94***	0.87***	1.00	–	–	–
	17. Maternal differential treatment	3.80 ± 0.85	−0.20***	0.38***	0.38***	−0.30***	0.33***	0.40***	0.93***	0.95***	0.99***	0.94***	0.95***	1.00	–	–
18. Self-esteem		2.96 ± 0.52	−0.07	0.10*	0.11*	−0.07	0.28***	0.25***	0.19***	0.20***	0.23***	0.23***	0.25***	0.23***	1.00	–
19. Internet addiction		0.27 ± 0.25	0.05	−0.11*	−0.11*	0.02	−0.15**	−0.12*	−0.24***	−0.22***	−0.23***	−0.22***	−0.20***	−0.23***	−0.39***	–

*P < 0.05, **p < 0.01, ***p < 0.001. Gender composition: brother-brother-1, sister-sister-2, brother-sister-3, sister-brother-4. Birth order: older sibling-1, younger sibling-2.

Gender was negatively correlated with self-esteem, boys had higher level of self-esteem. Age was negatively correlated with self-esteem and positively correlated with internet addiction. Birth order was negatively correlated with paternal and maternal PDT, as well as the fairness perception of paternal and maternal PDT, and significantly positively correlated with self-esteem. The results of the *T-*test found that older siblings scored higher on the PDT than younger siblings; that is, older participants were more favored. Older siblings scored higher on the fairness perception of PDT, that is, older siblings thought their parents were fairer. The self-esteem scores also showed a significant difference; older siblings had significantly lower self-esteem than younger ones.

### Moderated mediation model: paternal differential treatment

Table 2 presents results for the paternal model. First, the result of model 4 revealed that paternal PDT was negatively associated with IA. When self-esteem was included, paternal PDT was no longer associated with IA, but was positively associated with self-esteem, which in turn was negatively associated with IA. The indirect effect of paternal PDT on IA through self-esteem was −0.03 (95%CI [−0.05, −0.01]), indicating full mediation by self-esteem. This supports H2 for paternal PDT.

**TABLE 2:** Paternal differential treatment and internet addiction: a moderated mediation model.

Predictors	Self-esteem (step 1)		Internet addiction (step 1)		Self-esteem (step 2)		Internet addiction (step 2)	
	β	*t*	β	*t*	β	*t*	β	*t*
DTF	0.18	3.22**	−0.04	−1.40	0.08	0.39	0.16	1.80
PFF	–	–	−0.16	−6.68***	0.15	0.87	0.11	1.43
DTF × PFF	–	–	–	–	0.001	0.02	−0.06	−2.00*
R^2^	0.13		0.19		0.19		0.22	
F	9.24***		12.28***		10.40***		11.08***	

*p < 0.05, **p < 0.01, ***p < 0.001. DTF, paternal differential treatment; PFF, fairness perception of paternal differential treatment.

Then, the result of the moderating effect of fairness perception showed that the interaction of paternal PDT and fairness perception was significantly associated with IA (β = −0.06, *t* = −2.00, *p* < 0.05), indicating that fairness perception moderates the direct association between paternal PDT and IA (H4). Simple slope analysis (Figure 2) showed that when fairness perception was high (M + 1SD), paternal PDT was marginally negatively associated with IA (β = −0.10, *t* = −1.91, *p* = 0.057), suggesting a tentative trend that greater paternal favoritism was related to lower IA. However, when fairness perception was low (M−1SD), the association was non-significant (β = −0.001, *t* = −0.04, *p* > 0.05).

**FIGURE 2:**
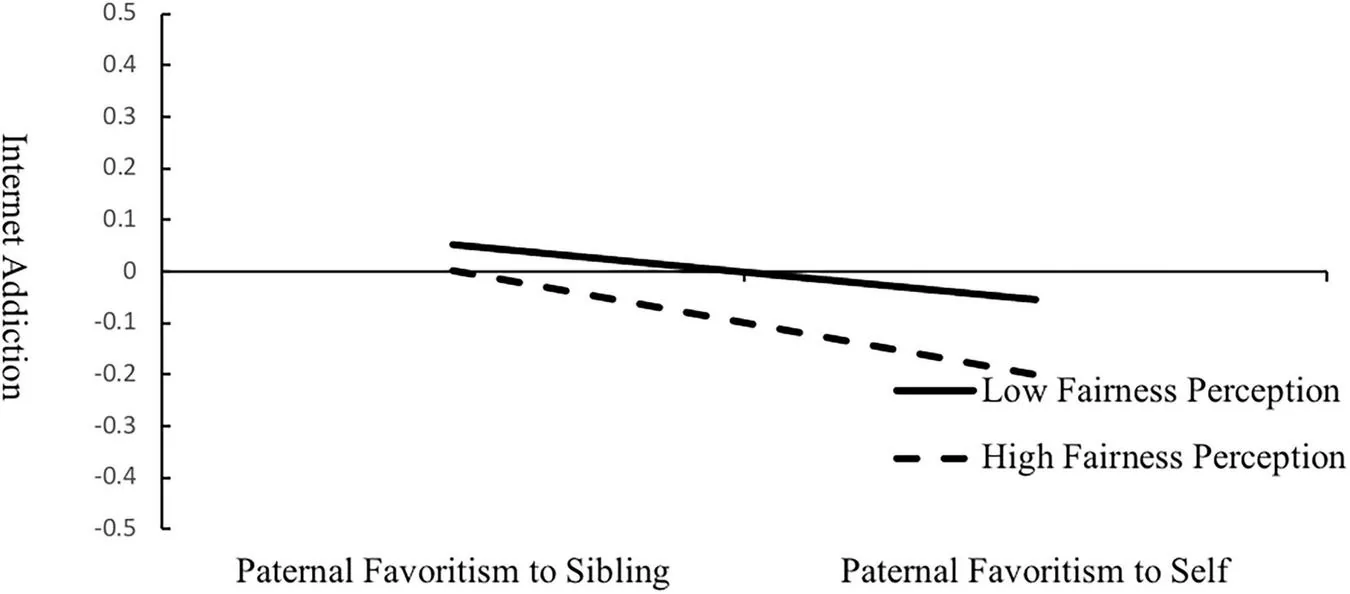
The moderating role of fairness perception on paternal differential treatment and internet addiction.

Lastly, the result of moderated mediation through self-esteem found that the interaction term of paternal PDT and fairness perception was not significantly associated with self-esteem, indicating that fairness perception does not moderate the indirect associated through self-esteem for paternal PDT. Thus, H3 was not supported for paternal PDT.

### Moderated mediation model: maternal differential treatment

Table 3 presents results for the maternal model. First, similar to the paternal model, maternal PDT was negatively associated with IA. When self-esteem was included, maternal PDT was no longer associated with IA but was positively associated with self-esteem, which was negatively associated with IA. The indirect effect was −0.05 (95% CI [−0.07, −0.03]), indicating a full mediating effect of self-esteem, supporting H2 for maternal PDT.

**TABLE 3:** Maternal differential treatment and internet addiction: a moderated mediation model.

Predictors	Self-esteem (step 1)		Internet addiction (step 1)		Self-esteem (step 2)		Internet addiction (step 2)	
	β	*t*	β	*t*	β	*t*	β	*t*
DTM	0.30	5.68***	−0.01	−0.27	−0.05	−0.25	0.14	1.65
PFM	–	–	−0.15	−6.24***	−0.12	−0.71	0.06	0.77
DTM × PFM	–	–	–	–	0.09	1.49	−0.04	−1.45
R^2^	0.19		0.18		0.23		0.21	
F	13.511***		11.23***		13.13***		10.48***	

*p < 0.05, **p < 0.01, ***p < 0.001. DTM, maternal differential treatment; PFM, fairness perception of maternal differential treatment.

Then, the interaction term of maternal PDT and fairness perception was not associated with IA and self-esteem.

## Discussion

### Perceived parental differential treatment and internet addiction

Consistent with H1, PDT (favoritism toward the self) was negatively correlated with adolescent IA. That is, adolescents who perceived relatively greater parental favoritism tended to report lower levels of IA. This finding aligns with Jensen and Whiteman (2014) observation that self-perceived favoritism is linked to fewer behavioral problems, extending this correlational pattern to IA.

Multiple psychological gains associated with perceived parental preference can explain this correlational pathway. Adolescents who sense they receive favored treatment from caregivers tend to maintain higher emotional security, robust offline social resources, and fuller gratification of fundamental psychological needs such as belongingness and competence (Deci and Ryan, 2000). These positive mental states are associated with lesser impulse to compensate for unmet needs via compulsive online engagement (Karaer and Akdemir, 2019). Conversely, adolescents who perceive less favoritism may use the internet to cope with feelings of neglect or rejection, as online environments provide opportunities for validation and escape (Ching and Tak, 2017). In the Chinese cultural context, this mechanism may be related to the allocation of emotional resources shaped by Fei Xiaotong’s differential mode of association. Adolescents receiving preferential parental treatment cultivate a stronger sense of family identity through sustained emotional investment from caregivers. By contrast, neglected siblings tend to suppress negative emotions, constrained by the prevailing cultural norm that prioritizes harmonious family relations. However, this affective suppression may be released indirectly through internet use, which may coincide with increased risk of addiction.

Notably, maternal PDT showed a stronger correlation with self-esteem, while paternal PDT’s association with IA was more pronounced in moderation analyses. Consistent with prior scholarly evidence, maternal and paternal parenting exert distinct gender-specific functions within families. Mothers typically serve as the primary providers of emotional nurturing, such that their differential affection correlates more strongly with adolescents’ self-esteem (Furnham and Cheng, 2000). Fathers, by comparison, carry greater weight in shaping youths’ behavioral self-regulation, which encompasses online engagement habits (Shek et al., 2019). In Chinese culture, where mothers are traditionally tasked with nurturing and fathers with discipline, these gendered effects are particularly salient (Luo et al., 2020). Furthermore, bivariate correlations indicated that the control dimension of PDT exhibited a positive correlation with birth order. In contrast, the affective dimension and total score demonstrated a negative correlation with birth order. Additionally, older children were more likely to report perceived favoritism. However, birth order itself did not correlate directly with the development of Internet addiction, suggesting that factors such as perceptions of fairness may moderate this relationship. This may be related to cultural norms of “ordering of elders and children,” which may lead to a higher tolerance of control among older children (Luo et al., 2020).

### The mediating role of self-esteem

Hypothesis 2 received full statistical support, as self-esteem acted as a complete mediator in the correlational pathway linking PDT and IA across maternal and paternal models. The result reveals that PDT correlates with adolescents’ internet addiction risk predominantly via shifts in their self-esteem. Perceiving parental preference correlates with elevated self-worth, and this positive self-perception further correlates with lower vulnerability to problematic online behaviors.

The positive association between PDT and self-esteem aligns with Pinquart and Gerke (2019) meta-analysis, which found that parental acceptance and affection are strong correlates of adolescent self-esteem. Favoritism signals parental approval, fostering a sense of self-worth and competence (Feinberg et al., 2000). For example, adolescents who receive more affection and less criticism from parents are likely to internalize these positive evaluations, developing higher self-esteem (Shebloski et al., 2005).

The negative link between self-esteem and IA replicates Ayas and Horzum (2013), who found that low self-esteem is correlated with increased IA. High self-esteem individuals are more resilient to stress and have greater confidence in their offline relationships, reducing their reliance on the internet (Andreassen et al., 2017). In contrast, low self-esteem adolescents may use the internet to escape negative self-perceptions or seek validation, which may coincide with increased risk of addiction (Shi et al., 2017).

Variations in self-esteem linked to family demographic composition warrant additional elaboration. For instance, boys raised alongside elder brothers or younger siblings tend to report more positive self-perceptions. This group of boys encounters less gender-biased PDT and gains robust support from same-gender siblings, factors that collectively correlate with lifted self-esteem levels (Kling et al., 1999). This finding aligns with the conclusions of Dan et al. (2019), which posits that, despite the presence of more stringent parental discipline in sibling pairs, the consistency in gender role expectations may serve to mitigate internal conflicts in self-perception. Younger siblings, despite older siblings reporting more perceived favoritism, reported higher self-esteem, possibly because older siblings bear greater family responsibilities (e.g., caring for younger siblings) and stress, which may undermine their self-esteem despite perceived parental favoritism (Suitor et al., 2017). This may also align with collectivism cultural norms that emphasize elder siblings’ caregiving roles (Luo et al., 2020).

### The moderating effect of fairness perception

Hypotheses 3 and 4 obtained partial statistical support, and the moderating role of perceived fairness presented divergent patterns across maternal and paternal PDT. The following sections separately unpack the moderating pathways observed in paternal and maternal caregiving contexts.

Turning to paternal PDT, perceived fairness functioned as a significant moderator shaping the connection between PDT and IA. Protective correlational patterns of paternal preferential care against problematic internet engagement only emerged when adolescents perceived this differential treatment as fair. This finding demonstrates that the favorable correlational patterns of paternal preference is conditional on adolescents’ subjective fairness judgment. If paternal favoritism is deemed inequitable, adolescents may experience intense guilt or escalated inter-sibling tension (Hamwey and Whiteman, 2021). Such negative emotional experiences may offset the potential benefits of paternal preference and may coincide with elevated risk of excessive online behavior. In comparison, adolescents who view paternal differential affection as equitable gain greater psychological security, which correlates with lesser urge to seek compensatory satisfaction through the internet (Karaer and Akdemir, 2019). Considering fathers’ established function in regulating adolescents’ externalized behaviors (Shek et al., 2019), paternal care shows stronger correlational protective patterns for internet addiction when adolescents accept such differential treatment as legitimate.

With regard to maternal PDT, fairness perception did not emerge as a significant moderator of the association between PDT and self-esteem. This finding stands in clear contrast to the significant moderating effect observed in the paternal PDT model, and the inconsistent patterns can be interpreted through differentiated parental socialization functions and parenting characteristics. Consistent with prior evidence, maternal parenting primarily correlates with adolescents’ internal self-evaluation, while paternal parenting is more strongly associated with external behavioral outcomes (Furnham and Cheng, 2000; Shek et al., 2019). Paternal differential treatment generally constitutes explicit resource allocation, yet its correlational association with internet-addiction risk is conditional on adolescents’ subjective fairness perceptions. In contrast, maternal differential treatment is embedded in daily, intimate emotional nurturing. Long-term maternal affective investment directly correlates with adolescents’ self-worth, and such stable internal self-perception is less susceptible to situational fairness evaluation. Accordingly, fairness perception functions as a critical boundary condition for paternal behavioral influence but exerts no observable conditioning effect on the linkage between maternal emotional nurturing and adolescent self-esteem.

Older siblings reported higher fairness perception, which may be consistent with cultural norms of “respecting elders and loving juniors” (Luo et al., 2020). They may rationalize PDT as necessary for younger siblings’ care, reducing perceived injustice (Suitor et al., 2017). This cultural context may highlight that fairness perception is not universal but shaped by normative beliefs about family roles, further underscoring the importance of cultural sensitivity in interpreting these findings. As Chen (2005) observes, fairness perception constitutes an individual’s subjective evaluation of the reasonableness of resource allocation. Within the context of the “equivocal” family ethic, differential treatment predicated on factors such as age or need is more likely to be perceived as a “reasonable difference” rather than an injustice.

## Conclusion

This study examined the correlational associations between PDT and adolescent IA, as well as the mediating role of self-esteem and the moderating role of fairness perception. The key findings are summarized as follows:

(1) PDT (favoritism toward the self) is negatively correlated with adolescent IA, indicating that adolescents reporting higher perceived self-favoritism tend to exhibit lower levels of IA.

(2) Self-esteem serves as a full mediator in the correlational pathway linking PDT and IA. Higher perceived PDT is correlated with elevated self-esteem, which further corresponds to lower IA levels among adolescents.

(3) Fairness perception yields no significant moderating effect on the association between maternal PDT and adolescent self-esteem after controlling for age, gender, sibling gender composition, age space and birth-order.

(4) Fairness perception significantly moderates the correlational relationship between paternal PDT and IA. The protective linkage between paternal self-favoritism and reduced IA only exists when adolescents report high fairness perception of paternal differential treatment.

## Limitations and implications

### Limitations

This study has several limitations that should be noted. First, the cross-sectional design precludes causal inference. While we hypothesize that PDT influences IA through self-esteem, it is also possible that IA reduces self-esteem, altering adolescents’ perceptions of PDT (e.g., feeling disfavored because parents disapprove of their internet use). Longitudinal studies are needed to test the directionality of these relationships.

Second, participants were recruited via convenience sampling from four northern and central Chinese provinces, so the sample is not nationally representative. The absence of western and southern participants may restrict generalizability due to regional differences in culture, socioeconomic status and family-rearing practices. In addition, despite sampling upper-primary, middle- and high-school students to cover early-to-late adolescence, developmental heterogeneity across age subgroups remains. We controlled for age as a continuous variable in all analyses, but age-stratified sensitivity analyses were not feasible given our sample size. Future studies should use larger, geographically diverse samples to verify model robustness and examine developmental variations.

Third, some questionnaires were excluded based on response quality. Excluded and retained participants differed in gender, age, school level and fairness perception, indicating non-random exclusion, which may introduce mild selection bias and limit generalizability of the findings.

Fourth, the use of self-report measures may introduce bias (e.g., social desirability, recall bias). For example, adolescents may underreport IA or over-report parental favoritism to present themselves more favorably. Multi-informant designs (e.g., including parent and teacher reports) would strengthen the validity of the findings.

Finally, this study draws primarily on Western theoretical frameworks and measures without fully incorporating Confucian-informed indigenous concepts such as differential order pattern and parent-child reciprocity. Western perspectives emphasize individual self-esteem in PDT processes, while Chinese adolescents may prioritize family identity, pointing to important cultural nuances. Relatedly, our Western-originated measures entail constraints. SIDE-C’s original scoring may not capture that strict discipline can reflect parental investment rather than disfavor in Chinese families. The derivative fairness-perception scale, one-to-one matched with PDT items, also lacks formal factor-structure evidence among Chinese adolescents; CFA-based item trimming was infeasible to preserve full PDT-behavior evaluations, requiring further validation.

### Theoretical implications

This study contributes to the literature in several ways. First, it extends research on parenting and IA by focusing on PDT, a comparative parenting practice that is particularly relevant in multi-child families. By demonstrating that PDT (favoritism toward the self) is associated with lower IA, we highlight the importance of considering sibling dynamics in understanding adolescent internet use.

Second, we identify self-esteem as a key mediator, shedding light on the psychological mechanism linking PDT to IA. This supports social comparison theory (Festinger, 1954) and self-determination theory (Deci and Ryan, 2000), showing that parental favoritism is associated with self-evaluation, which in turn relates to adaptive or maladaptive behaviors.

Third, we highlight the moderating role of fairness perception, emphasizing that the correlational patterns linked to PDT depend on subjective evaluations rather than objective treatment alone. This aligns with equity theory (Hatfield et al., 1978) and underscores the importance of adolescents’ cognitive appraisals in shaping the impact of family processes.

Finally, by distinguishing paternal and maternal effects, we contribute to understanding the gendered nature of parenting in Chinese culture, where mothers and fathers play distinct roles in shaping emotional and behavioral outcomes (Furnham and Cheng, 2000; Shek et al., 2019). Within the context of Chinese culture, this suggests culturally rooted perceptions of “reasonable differences” (e.g., interpretations based on birth-order norms and tailored parenting) may act as a protective factor against the adverse correlates of PDT.

### Practical implications

The present results carry meaningful practical implications for parents, educational practitioners, clinical counselors and policymakers who serve multi-child households.

Interventions targeting family parenting practices constitute the most direct application of this study’s conclusions. Empirical evidence confirms that nurturing adolescents’ self-esteem and cultivating their sense of fair treatment may coincide with lower likelihood of problematic internet use. Caregivers are encouraged to reconcile inevitable differentiated care with transparent communication within the household. Clarifying the reasoning behind uneven arrangements, such as offering extra academic assistance to younger children due to their developmental limitations, helps adolescents recognize parental arrangements as reasonable (Zhang and Cao, 2021). Parents ought to avoid direct comparative remarks between siblings, a behavior widely documented to trigger negative emotional experiences, especially amid gaps in gender and birth order. Family care should integrate the traditional balance of hierarchical order and impartiality while acknowledging each child’s distinct developmental demands (Chen et al., 2017). Delivering personalized emotional care and support to every sibling according to individual traits may correlate with stronger adolescents’ self-worth and lessen the tendency to seek emotional compensation online.

Beyond domestic settings, school educators and mental health practitioners may draw targeted intervention strategies from the research data. Youths with low self-esteem represent a high-risk group correlated with internet addiction, making self-esteem promotion a core intervention priority. Training sessions centered on competency cultivation and constructive feedback serve as viable approaches that may relate to reduced excessive online engagement. Family counseling also facilitates open dialogue between parents and children regarding differentiated care, which may correlate with improved adolescents’ fairness judgements and eases inter-sibling friction. Specialized counseling programs can be developed for former only children adjusting to the role of elder siblings, easing feelings of loss stemming from shifts in family resource allocation after a second child joins the household.

Policy design represents a broader dimension for translating research evidence into real-world support systems. Authorities can launch universal parenting advocacy initiatives tailored to multi-child families, highlighting the dual importance of consistent emotional care and perceived equity in parental treatment. Public resources including offline parenting seminars and digital guidance materials equip caregivers to address common challenges of raising multiple children, which may correlate with lower prevalence of internet addiction and other externalizing behavioral issues. Local family education guidance may integrate traditional cultural narratives like the story of Kong Rong yielding larger pears to illustrate how rational differential care maintains stable and harmonious family dynamics while respecting Chinese cultural values.

## Data Availability

The raw data supporting the conclusions of this article will be made available by the authors, without undue reservation.
